# Analysis of HIV-1 Protease Gene Reveals Frequent Multiple Infections Followed by Recombination among Drug Treated Individuals Living in São Paulo and Santos, Brazil

**DOI:** 10.1371/journal.pone.0084066

**Published:** 2014-01-03

**Authors:** Edsel Renata De Morais Nunes, Jean Paulo Zukurov, Juliana Terzi Maricato, Maria Cecília Araripe Sucupira, Ricardo Sobhie Diaz, Luíz Mário Ramos Janini

**Affiliations:** 1 Department of Medicine, Federal University of São Paulo, São Paulo, Brazil; 2 Department of Microbiology, Immunology and Parasitology, Federal University of São Paulo, São Paulo, Brazil; Centers for Disease Control and Prevention, United States of America

## Abstract

The present study investigated the prevalence of HIV-1 multiple infections in a population composed by 47 patients under HAART failure and enrolled at the National DST/AIDS, Program, Ministry of Health, Brazil.Detection of multiple infections was done using a previously published RFLP assay for the HIV-1 protease gene, which is able of distinguishing between infections caused by a single or multiple HIV-1 subtypes. Samples with multiple infections were cloned, and sequence data submitted to phylogenetic analysis. We were able to identify 17 HIV-1 multiple infections out of 47 samples. Multiple infections were mostly composed by a mixture of recombinant viruses (94%), with only one case in which protease gene pure subtypes B and F were recovered. This is the first study that reports the prevalence of multiple infections and intersubtype recombinants in a population undergoing HAART in Brazil. Based on the data there was a steep increase of multiple infections after the introduction of the combined antiretroviral therapy in Brazil. Cases of multiple infections may be associated with HIV-1 genetic diversity through recombination allowing for the generation of viruses showing a combination of resistance mutations.

## Introduction

According to the UNAIDS there are more than 34 million people living with HIV-1. The global distribution of HIV-1 infections is not uniform. The Sub-Saharan Africa accounts for 23.5 million infections being the most prevalent region [1]. HIV-1 subtype distribution is not balanced. There are regions affected by mostly one predominant viral variant while others demonstrate the simultaneous circulation of more than one subtype [2]. Subtype B dominates the HIV-1 epidemic in Western Europe, and in the USA. On the other hand, almost all subtypes have been described in Central Africa [3], [4]. The co-circulation of more than one HIV-1 genetic form in the same population may lead to multiple viral infections, which may offer an opportunity for viral recombination [3]. Recombination is an important feature of the HIV-1 pandemics. There are 58 distinct HIV-1 circulating recombinant forms (CRFs) posted on the Los Alamos HIV-1 Database as of January 2013 [5]. Although some of these recombinant forms are composed by a mixture of two different subtypes others have more than two genetic origins and are referred to as complex recombinants. Is it also noteworthy that a few CRFs may represent a second generation of recombinant forms since they bear in their structures segments belonging to other previously described CRFs. CRFs represent 20% of all HIV-1 infections worldwide [2], [6]. CRF01_AE and CRF02_AG account for approximately 3.6 million infections. Moreover, in places where more than one HIV-1 subtype circulates, 30% of the infected individuals may carry HIV-1 unique recombinant forms [2], [7]. The high prevalence of recombinants suggests that multiple infections may not be rare [8]–[13]. Multiple infections result from either coinfections (acquisition of two distinct viruses at the same time) or superinfections (sequential acquisition of distinct viruses). Superinfections may result in the acquisition of drug-resistant isolates [14] and accelerated disease progression [15]. A study conducted in 2010 with elite controllers found that superinfections were associated with disease progression [16]. Gottlieb and colleagues [17] studying four cases of HIV-1 coinfections and one superinfection noted that infections caused by mixed virus populations evolved at an accelerated pace to clinical AIDS or to TCD4 counts of less than 200/mm3 when compared to single infections. However, Fung and colleagues [18] using a mathematical model inferred that superinfections do not trigger or accelerate progression to disease.Some studies noted that prolonged highly active antiretroviral therapy (HAART) may influence the immune response [19], [20]. It is believed that long term viral suppression may affect antibody titers and the lack of neutralizing antibodies may favor superinfections [20]. However, Piantadosi and colleagues [21] reported several cases of HIV-1 superinfections in which patients were not under HAART. The study of HIV-1 multiple infections is essential in areas where multiple HIV-1subtypes co-circulate. As mentioned above, multiple infections may increase the genetic complexity of virus populations [22]. Furthermore, superinfections may interfere with the HAART success and affect disease progression by the sequential acquisition of viruses with different resistance associated mutations [14], [23].

HIV-1 multiple infections caused by viruses from the same or different subtypes have been previously reported [10], [13],[17],[20],21],[24]–[42],[43]–[49]. This type of infection has been mostly identified in populations at high risk of becoming infected by HIV-1. These populations correspond to groups of inject drug users (IDUs), men who have sex with men (MSM), and sex workers. HIV-1 multiple infections ranged from 1.4% to 19% among African sex workers [17], [21], [33], [37]–[39], [50]. It also accounted for 25% of all HIV-1 cases in a population of MSM in the USA [30]. Of note, in 2007 a study addressing a criminal case involving 14 newly infected MSM in Holland demonstrated the presence of multiple infections in half of the subject individuals [27]. HIV-1 multiple infections varied from 1.5% to 6% among IDUs in Thailand [35], [42], [48]. Templeton and co-workers [28] found that multiple infections corresponded to 40% of the total number of HIV-1 infections in a population of IDUs enrolled in a 6 year study (1994–2000). In populations without a defined risk of exposure, multiple infections were detected from 8% to 15% from the total HIV-1 studied cases [25], [26], [10].

In South America, the majority of studies have been done addressing populations in Brazil and Argentina. A study from 2000 detected 1.7% of HIV-1 multiple infections in Buenos Aires [44]. In 2007, while addressing a population of IDUs in Argentina, Pando et al. [32] found that multiple infections corresponded to 6% of all studied cases. In a recent investigation [24] conducted on a group of 23 Argentinean patients at a high HIV-1 exposure risk, multiple infections were detected in 5 of them. Four of these infections were composed by subtype B and BF recombinant viruses. The fifth multiple infection had only BF recombinants.

In Brazil several HIV-1 subtypes co-circulate. The first subtype introduced in Brazil was most likely subtype B followed by subtypes F and C. In regions such as the Southeast, the presence of more than one circulating subtype maybe is related to the detection of recombinant forms. The majority of these recombinants correspond to unique recombinants most likely arising from different multiple HIV-1 infection events [51]–[53]. Furthermore, six CRFs have been described in Brazil [54]–[57]. Since the late nineties, the Brazilian Ministry of Health has been funding the distribution of antiretroviral treatment.

The first studies on dual infections in Brazil were done prior to the introduction of HAART. In the 1990s, Janini and coworkers [47] and Ramos et al [45] found that multiple infections corresponded respectively to 3 and 3.8% when studying population groups in Brazil. The combination between the co-circulation of multiple subtypes and a broadly accessible HAART might have created the necessary conditions leading to an increase in multiple infections. This study aimed to assess the prevalence of multiple infections in patients experimenting virological failure while under HAART and living in the Southeast of Brazil. We used as trial a previously published restriction length polymorphism assay (RFLP) based on the HIV-1 protease gene digestion patterns [47]. RFLP data was confirmed by cloning, sequencing and phylogenetic trees. Multiple infections corresponded to 36% of all HIV-1 infections in the studied subjects. All but one multiple infections showed the presence of recombinant viruses. This data indicates that multiple infections may be taken into account as a driving force increasing the genetic complexity of the HIV-1 epidemic in the Southeastern region of Brazil.

## Materials and Methods

The study was Institutional Review Board approved at the Federal University of Sao Paulo, Brazil, and written informed consent has been obtained (process number in research ethics committee 0075/09).

### Studied population

Peripheral blood samples were obtained from 47 HIV-1 infected individuals living in the cities of Sao Paulo and Santos and enrolled at the governmental program of Sexually Transmitted Diseases/AIDS (DST/AIDS), Ministry of Health – Brazil. Samples were collected during 2006 and 2008. Patients were under highly active antiretroviral treatment according to the Brazilian Government Guidelines for Antiretroviral Therapy.

### Polymerase chain reaction (PCR)

Viral DNA was extracted and used as target to amplify a 297-bp fragment corresponding to the HIV-1 protease gene. Nested PCR conditions and the set of primers used were described by Janini et al. [47].

### Restriction fragment length polymorphism analysis (RFLP)

Restriction enzyme reactions consisted of 8 uL of the PCR product corresponding to the amplified viral protease gene, 3U of AluI restriction enzyme (Invitrogen), and 1 mL of enzyme buffer supplied by the manufacturer. Digestions were performed according to Janini et al. [47]. After incubation at 37°C for 2 hours, digested products were analyzed by agarose gel (1%) electrophoresis and stained with SYBER® Safe DNA Stain 10.000X (Invitrogen). Positive controls representing different HIV-1 subtypes were included to provide known restriction fragment patterns.

### Cloning and Sequencing

PCR-amplified proteasesequences were cloned into thepDrive cloning vector using the QIagen PCR Cloning Kit (QIAGEN Inc.). A minimum of 12 and a maximum of 70 clones were obtained from each sample in order to isolate sequences with distinct phylogenetic origins. Clones were screened by the protease gene RFLP assay. Sequence reactions were performed using the Big-Dye Terminator v3.1 Cycle Sequencing (Applied Biosystems), according to the manufacturer's instructions and analyzed using the ABI 3130 xl Genetic Analyzer Program (Applied Biosystems).

### Analysis of Results

Sequences were assembled using the Sequencher software version 4.1.4 (Gene Codes Corporation). Resulting sequences were manually edited with BioEdit version 7.0.0 (Ibis Biosciences, USA) [58]. Protease sequences were aligned using the program Muscle V3.8.31 [59].

### Phylogenetic Inference

Phylogenetic inference was performed using Paup 4.0 Beta [60] in association with Modeltest version 3.7 [61]. Phylogenetic analysis was performed using the Neighbor Joining (NJ) method and the TVM + Γ was the most appropriate nucleotide substitution model. Constructed trees were checked by 1000 bootstrap replicates. Trees were edited using the software Figtree [62].

### Detection of Recombination

Inter-subtype recombinants and breakpoints were investigated using Bootscan as implemented in the Simplot package v3.5.1 [63]. Bootscananalysis was based on the Neighbor-Joining algorithm with the K2P model and was evaluated by 1000 bootstrap replicates. The analysis was performed with an 75nt window and 5 nt increments.

## Results

**Figure 1:**
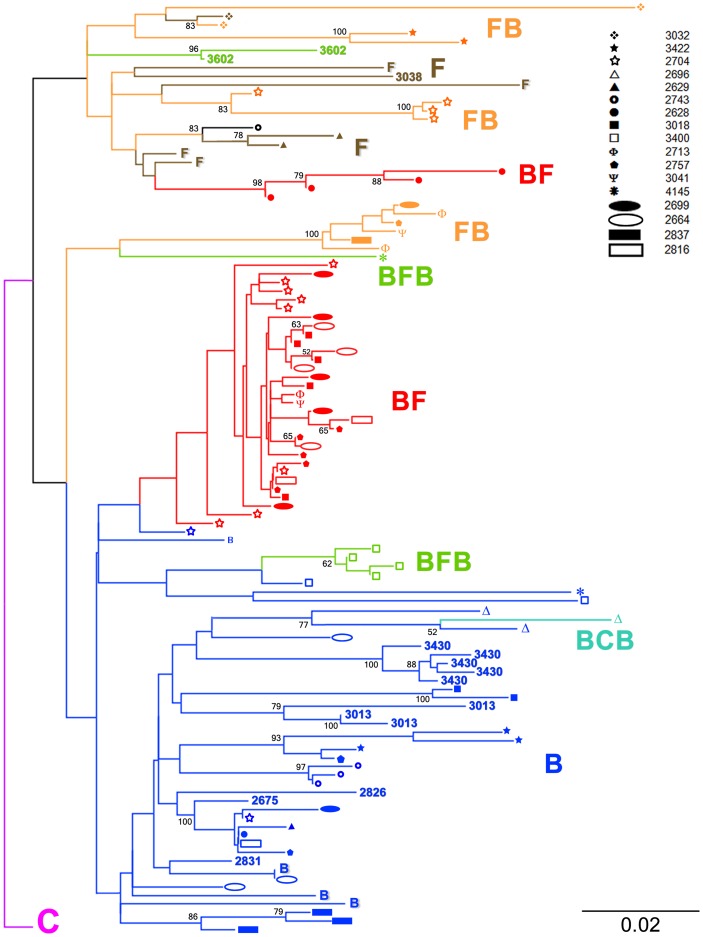
Protease gene phylogenetic classification of HIV-1 multiple infections. Multiple infections are represented on the tree by different branches showing the same icon at their tips. Samples 2675, 2831, 2826, 3013, 3038, 3430 and 3602 correspond to simple infections. The NJ tree was built using TVM + Γ as the nucleotide substitution model.

We addressed samples from HAART experienced patients under virological failure and enrolled at the governmental program of Sexually Transmitted Diseases/AIDS (DST/AIDS), Brazil. Using a combination of protease gene RFLP assay followed by cloning and sequencing analysis we were able to identify 17 HIV-1 multiple infections out of 47 tested samples. These multiple infections were composed by mainly subtype B and F protease sequences and BF recombinants with one case of a BCB recombinant. In our study all but 2 subtype F protease gene sequences were in fact BF recombinants. Samples showing multiple protease gene RFLP digestion patterns were cloned and sequenced. Sequences from all 17 multiple infections and 7 from simple infections were used along protease gene subtype specific reference strains obtained from GenBank to build a NJ phylogenetic tree. (GenBank accession numbers: subtype B references from Brazil AY173956, EF637057, from California DQ322225 and from Argentina DQ383748; subtype C from Brazil GU982733; subtype F from Brazil AY900894, FJ591905 and sub-subtype F1 from Brazil DQ899684 and FJ405153). This tree is shown in Figure 1. Sequences generated in this study were granted the GenBank accession numbers KF767573– KF767672.

Pure subtype B protease gene sequences in dark blue formed a major and well defined cluster seen at the bottom of the tree. Pure subtype F sequences (in brown) interspersed by FB (orange), BF (red) and BFB (green) recombinants were grouped at the top of the tree forming an isolated cluster emerging from a single ancestral node. The protease gene sequence obtained from the sample 2743, which was a recombinant between an unclassified segment and subtype F, also grouped within this cluster.

Two other groups of sequences composed by exclusively recombinant protease gene sequences can be seen at the middle of the tree. First, an isolated group containing only FB and BFB recombinants and a second cluster formed by BF recombinants emerging from a branch contained within the pure subtype B cluster. The emergence of the above mentioned BF cluster as well as other minor clusters as the BFB group of sequences and the BCB recombinant sequence (light blue) from within the pure subtype B group implies a phylogenetic relatedness of these recombinants with pure subtype B protease gene sequences used here. The overall placement of recombinants in the tree was strongly influenced by their subtype content. As an example, BFB recombinants from samples 3400 and 4145 differed in their subtype F content by only 14 nucleotides what was sufficient to grant their separation in two distant clusters. In the tree multiple infections are represented by distinct branches showing geometric symbols at their tips. Each multiple infection is represented by a unique symbol. By following the symbols it is clear that sequences from multiple infected patients grouped within different clusters, enabling us to infer that each patient carried an infection composed by phylogenetically distinct virus (Figure 1). Single infections are represented in the tree by sample numbers. Samples 3032, 3422, 2704, 2696, 2629, 2743, 2628, 3018, 3400, 2713, 2757, 3041, 4145, 2699, 2664, 2837 and 2816 corresponded to the 17 multiple infections detected in our study while samples 2675, 2831, 2826, 3013, 3038, 3430 and 3602 were HIV-1 single infections.

The Bootscan analysis confirmed the genetic composition of the 17 HIV-1 multiple infections by demonstrating that the majority of multiple infections were composed by a combination of pure subtype B protease sequences and recombinants. According to the Bootscan analysis multiple HIV-1 infections corresponded to 3 triple infections composed each one by the simultaneous presence of viruses containing pure B subtype protease gene sequences and BF and FB recombinants; 4 double infections composed by pure subtype B viruses and BF recombinants; 2 double infections composed by pure subtype B and FB recombinants; 2 double infections composed by BF and FB recombinants; 2 double infections composed by pure subtype B and BFB recombinants; 1 double infection composed by pure subtype B and a BCB recombinant; 1 double infection composed by pure subtype F and a FB recombinant; and one double infection composed by protease genes from pure B and F subtypes.

**Figure 2:**
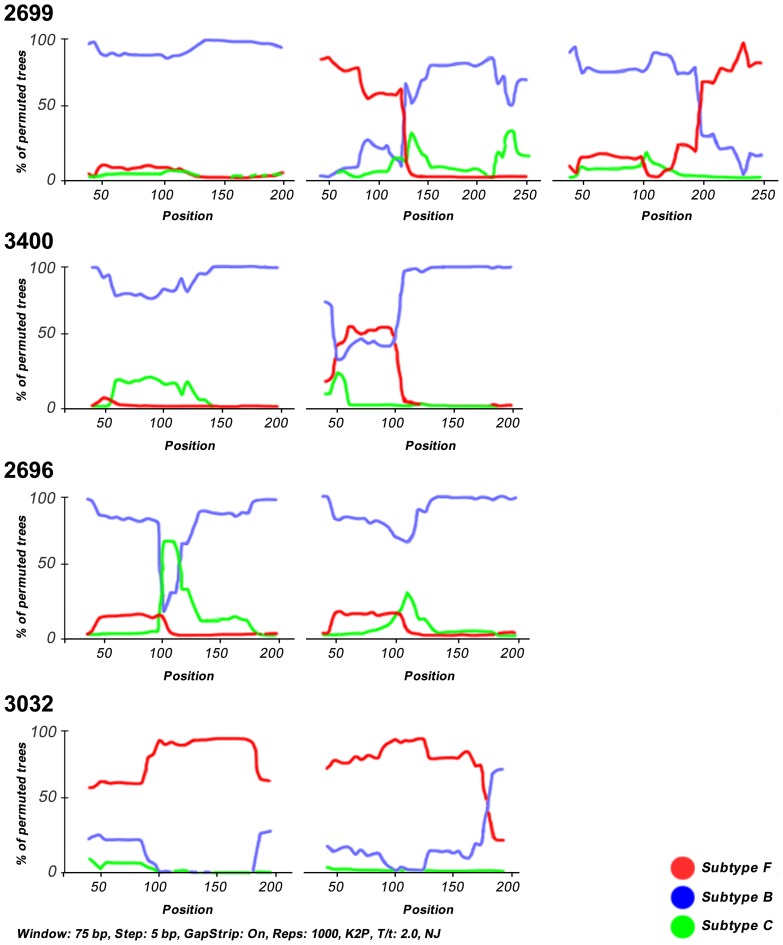
Bootscan analysis of HIV-1 multiple infections based on the protease gene. We used the Neighbor-Joining method with K2P and 1000 bootstrap replicates. This analysis was performed using the software Simplot v3.5.1, with a window of 75 bp and 5 bp increments.

Among recombinants, BF mosaics generally predominated over the other recombinants. The second most prevalent mosaics were FB recombinants followed by BFB structures, 1 BC recombinant and 1 unclassified/F strain. Breakpoints were defined as the midpoint of an interval flanked by two distinct phylogenies. Breakpoints and recombination intervals can be seen in table 1. Although the criteria used to assign a subtype to any sequence was based on a 70% bootstrap value threshold, in the sample 3400 the subtype F sequence stretch had a bootstrap value close to 50% (Figure 2). However BFB recombinants placed distantly from pure subtype B protease gene sequences from this patient in the tree (Figure 1) indicating this was a real HIV-1 multiple infection case.

**Table 1:** Breakpoints and Recombination Individuals.

Samples	Subtype	RecombinationInterval	Breakpoint (BP)
**2628**	B/F	50–78	64
**2628**	B/F	58–82	70
**2628**	B/F	58–82	70
**2628**	B/F	65–82	73
**2664**	B/F	182–190	186
**2664**	B/F	184–188	186
**2664**	B/F	184–188	186
**2664**	B/F	183–189	186
**2696**	B/C/B	141–145/155–159	143/157
**2699**	F/B	127–131	129
**2699**	B/F	188–220	204
**2699**	B/F	186–220	203
**2699**	B/F	185–189	187
**2699**	B/F	186–220	203
**2699**	B/F	186–202	194
**2704**	B/F	76–81	78
**2704**	B/F	60–79	69
**2704**	B/F	60–79	69
**2704**	B/F	60–79	69
**2704**	B/F	185–189	187
**2704**	B/F	185–189	187
**2704**	B/F	171–207	189
**2704**	B/F	174–204	189
**2704**	B/F	188–220	204
**2704**	B/F	170–208	189
**2704**	B/F	188–220	204
**2704**	B/F	185–193	189
**2713**	B/F	139–151	145
**2713**	B/F	137–153	145
**2713**	B/F	188–220	204
**2757**	B/F	186–190	188
**2757**	B/F	186–190	188
**2757**	B/F	143–147	145
**2757**	B/F	186–200	193
**2757**	B/F	186–190	188
**2757**	F/B	170–174	172
**2816**	B/F	186–190	188
**2816**	B/F	186–190	188
**2837**	F/B	141–159	150
**3018**	B/F	185–189	187
**3018**	B/F	185–189	187
**3018**	B/F	185–189	187
**3018**	B/F	185–189	192
**3018**	B/F	185–189	187
**3032**	F/B	219–227	223
**3032**	F/B	218–228	223
**3041**	F/B	135–157	146
**3041**	B/F	185–189	187
**3400**	B/F/B	84–100/138–142	92/140
**3400**	B/F/B	84–100/138–142	92/140
**3400**	B/F/B	83–101/133–147	92/140
**3400**	B/F/B	84–100/138–142	92/140
**3422**	F/B	219–223	221
**3422**	F/B	218–224	221
**4145**	B/F/B	88–93/138–166	93/152

Figure 2 shows Bootscan illustrations from 4 representatives HIV-1 multiple infections. In the top panel patient 2699 harbors a triple infection composed by virus belonging to pure subtype B and FB and BF recombinants. In the top middle panel, patient 3400 shows a HIV-1 double infection by pure subtype B virus and BFB recombinants. The lower middle panel shows a case of a double infection by pure subtype B virus and a BCB recombinant (patient 2696) and in the lower panel one double infection by pure subtype F and FB recombinants (patient 3032).

**Figure 3:**
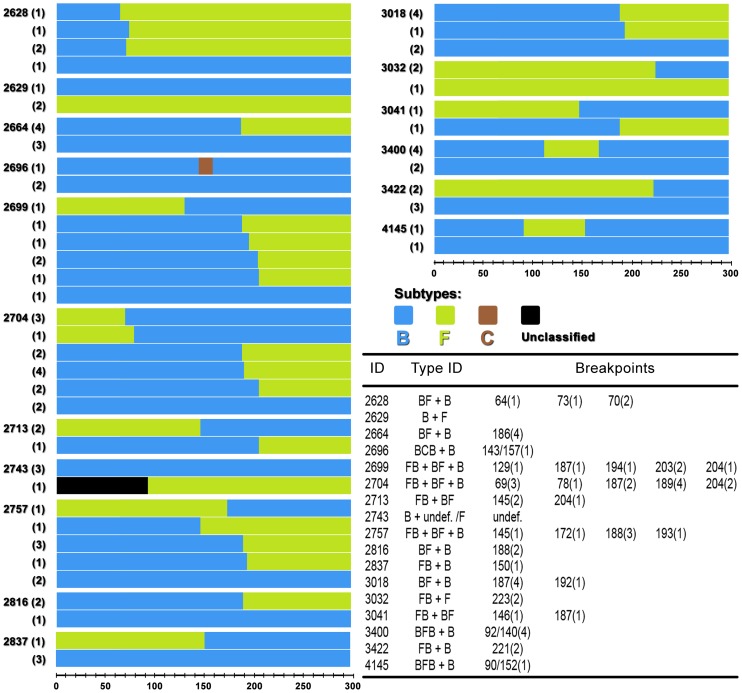
Schematic mosaic structures and pure subtypes found in multiple infections. The number of times that a particular subtype or recombinant was found in a patient is shown inside the parenthesis to the left of each structure. Break points were mapped according to Bootscan and their positions in base pairs are followed by the number they appeared on mosaic structures (inside the parenthesis).

In Figure 3 schematic mosaic structures of recombinants and pure subtypes found in each patient are represented. The number of times that a particular subtype or recombinant form was found in a patient is shown inside the parenthesis to the left of each structure. In the table within the figure, breakpoint positions in base pairs are followed by the number of times they appear in each multiple infection (inside the parenthesis). It is clear that according to the mosaic schemes majority of recombinants were patient specific.

**Figure 4:**
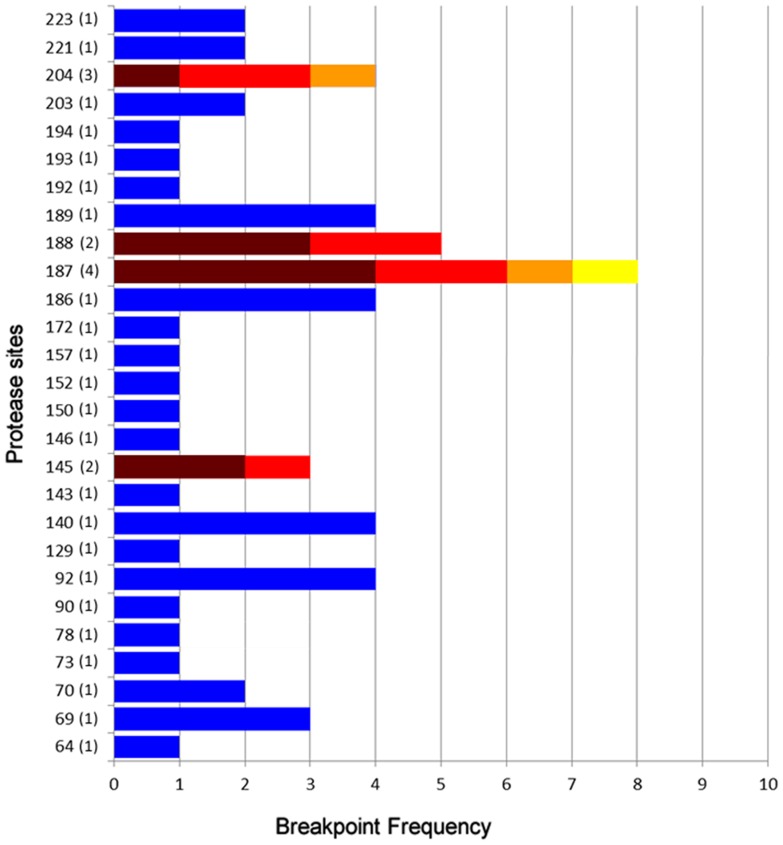
Protease gene break point positions in nucleotides and their frequency. X axis indicates the number each breakpoint was detected in our study. Break points detected in single patients are represented by Y axis bars in blue whereas; break points detected in multiple patients appear in multicolored bars. The numbers inside parenthesis are the number of different patients in which this particular break point was detected.

Breakpoints were distributed all over the protease gene sequence indicating the absence of clear recombination hotspots among the mosaic sequences. However, it is interesting to note that the same recombination breakpoint appears more than once in a patient, such as in patient 2628 and also between different patients as in patients 2699, 2704 e 3018. Breakpoint distribution and times they appear along the protease gene are represented in Figure 4.

## Discussion

This study addressed the presence of HIV-1 multiple infections in samples obtained from patients during HAART failure. Using a previously published protease gene RFLP assay followed by cloning and sequencing we were able to identify 17 HIV-1 multiple infections out of 47 samples. Multiple infections corresponded to 36% of all HIV-1 infections among the studied subjects. The percentage of HIV-1 multiple infections found here is between 9 to 12 times greater than the percentages encountered in studies by Janini et al [47] and Ramos et al. [45]. These studies were conducted in Brazil in the early nineties before the introduction of HAART. Although some publications indicated that HIV-1 multiple infections could be detected in up to 40 to 50% of studied cases, most of those investigations addressed particular situations or were associated with populations exposed to risky behavior, with multiple exposures through the sexual route or by intravenous drug use [27], [28], [35], [40], [42], [48].

In contrast, we found an elevated frequency of multiple infections in a population that was not uniformly composed by individuals at an increased risk of HIV-1 exposure. The increased number of multiple infections among patients living in the cities of São Paulo and Santos may be reflecting aspects of the epidemic in the Southeast region of Brazil. Among these are the co-circulation of distinct subtypes and recombinants and the presence of an efficient and tight transmission chain. The Southeast region of Brazil, where the city of São Paulo is located, has the majority of the HIV-1 cases in the country [53], [66]. Because some studies have addressed HIV-1 superinfections in treatment experienced patients, [14], [19] it is tempting to speculate that the observed increase of HIV-1 multiple infections also holds a relationship with the introduction of HAART in Brazil. Since the late nineties the Brazilian Government has been applying funds making HAART broadly accessible to Brazilian HIV-1 infected individuals. Long term suppression of virus replication might favor superinfection by drug resistant variants resulting in increased numbers of multiple infections among patients experiencing treatment failure.

Although we could successfully identify many cases of multiple infections, the study of this type of infections can be difficult. Approaching clinical samples by direct sequencing without cloning may result in failure to detect minor viral populations which might become predominant at a later time [70]. The application of a trial methodology prior to sequencing may facilitate sorting HIV-1 multiple infections from single infections. We believe that by using a previous described RFLP assay based on the HIV-1 protease gene digestion patterns our power to detect multiple infections was increased [47]. The simultaneous presence of more than one protease gene digestion patterns was used as indicative of multiple infections. Samples with multiple digestion patterns were subsequently cloned and sequenced. Moreover, the protease gene is a fairly conserved gene what facilitates PCR detection and according to several previous publications [67]–[69], proved to be a gene with sufficient phylogenetic signal to classify samples.

The elevated frequency of HIV-1 multiple infections described here suggests that the genetic complexity of the HIV-1 epidemic in at least the Southeastern region of Brazil is indeed increasing.

One of the outcomes of HIV-1 multiple infections is the recombination between distinct viral populations [2]. Recombination can lead to evolutionary leaps and facilitate the emergence of particles with different phenotypic characteristics and may accelerate the process of viral adaptation to a host or host population [64], [65]. Lately, a steady increase in the numbers of reported HIV-1 recombinant strains have been observed in the Brazilian epidemic [54]–[57]. In this scenario, it is acceptable to think that the increasing numbers of multiple infections has been fuelling the generation of recombinant strains in Brazil.

From the 17 multiple infections detected in our study, 16 involved the presence of recombinants. According to the protease gene, only one multiple infection was composed by pure subtype viruses. In this case one virus population belonged to subtype B and the other to subtype F. Of the remaining 16 multiple infections, 14 were composed by viruses with their protease genes showing a mixture of subtypes B and F along with viruses that had the protease gene classified as either pure subtype B or pure subtype F. Of the two remaining multiple infections, one was composed by a combination of subtype B and BC recombinant viruses, and the other by a mixture of pure subtype B and an undefined/F recombinant. Once more, our findings are in agreement with the distribution of HIV-1 subtypes in the Brazilian epidemic. The Southeast is a region where subtypes B and F co-circulate, in this manner recombinants between these two subtypes can be expected [53], [66].

After the analysis of recombinant sequences obtained from multiple infections, it became clear that they corresponded to a full spectrum of BF recombinants. Recombinants ranged from mostly subtype B to mostly subtype F with many of them showing different percentages of subtypes B and F to their structures. This spectrum can be easily visualized in Figure 3. The tree topology demonstrates distinct clusters of recombinants, with groups formed by recombinants with more subtype B than F (red cluster) and with more subtype F than B (orange sequences). This observation suggests that subtypes B and F are getting mixed with each other through frequent multiple infections followed by recombination. As mentioned before, in one sequence the 5′ portion of the protease gene remained unclassified. It is possible that the intense mixing of subtypes B and F with the presence of multiple breakpoints may have rendered this fragment unclassifiable. The intense mixture of subtypes B and F isn't new to the South American epidemic [2]. Several distinct CRFs with subtypes B and F have been described. The South American BF CRFs are composed by different portions of subtypes B and F [2], [5]. Our data support the idea that frequent multiple infections followed by recombination are helping subtypes B and F to merge in South America. As an example, we were able detected three triple infections being all composed by subtype B virus, BF and FB recombinants.

The Bootscan analysis demonstrated recombinants corresponded to different BF mosaics. Many of these mosaics showed patient specific patterns, suggesting the presence of intrapatient recombination. On the other hand, detection of some BF forms showing a very similar mosaic structure in more than one patient indicated that some recombinants may represent a still not well defined CRF expanding in the study population. This CRF could be a component of many multiple infections. However, we found no correlation between our recombinant patterns and the already described CRFs in the HIV-1 epidemic in Brazil (data not shown). Since the addressed genomic region in our study corresponded to the protease gene we were unable to make any assumptions regarding the genomic structure of these recombinants or of putative CRFs.

Finally, in the present study we describe a high percentage of HIV-1multiple infections in samples from individuals undergoing HAART and experiencing treatment failure. Long term viral suppression might offer an opportunity to superinfection caused by drug resistant virus [14], [71]–[73]. However, to access the distribution of multiple infections in Brazil and its correlations with HAART other population groups need to be approached. We were able to detect many distinct recombinants as part of multiple infections. Further studies using longer subgenomic segments or full length HIV-1 genomes might reveal an even greater number of HIV-1 recombinants in the Southeastern region of Brazil.
